# Scaling Up Latent Tuberculosis Infection Testing and Treatment for Non-US Born Patients in a Federally Qualified Community Health Center

**DOI:** 10.1007/s10903-023-01514-0

**Published:** 2023-07-10

**Authors:** J. Cochran, A. Tibbs, H. H. Haptu, R. K. Paradise, J. Bernardo, D. B. Tierney

**Affiliations:** 1 Massachusetts Department of Public Health, Boston, MA USA; 2 Lynn Community Health Center, Lynn, MA USA; 3 Institute for Community Health, Malden, MA USA; 4 Tufts University School of Medicine, Boston, MA USA; 5 Boston University School of Medicine, Boston, MA USA; 6 Brigham and Women’s Hospital, Boston, MA USA

**Keywords:** Tuberculosis, LTBI, Community health center, Non-US born

## Abstract

In the United States (US), tuberculosis elimination strategies include scaling up latent tuberculosis infection (LTBI) diagnosis and treatment for persons at risk of progression to tuberculosis disease. The Massachusetts Department of Public Health partnered with Lynn Community Health Center to provide care to patients with LTBI who were born outside the US. The electronic health record was modified to facilitate collection of data elements for public health assessment of the LTBI care cascade. Among health center patients born outside the US, testing for tuberculosis infection increased by over 190%. From October 1, 2016 to March 21, 2019, 8827 patients were screened and 1368 (15.5%) were diagnosed with LTBI. Using the electronic health record, we documented treatment completion for 645/1368 (47.1%) patients. The greatest drop-offs occurred between testing for TB infection and clinical evaluation after a positive test (24.3%) and between the recommendation for LTBI treatment and completion of a treatment course (22.8%). Tuberculosis care delivery was embedded in the primary care medical home, bringing patient-centered care to those at high risk for loss to follow up. The partnership between public health and the community health center promoted quality improvement.

## Background

Tuberculosis (TB) disease caused 1.5 million deaths globally in 2020 [1]. In the United States (US), the burden of TB disease is concentrated in populations born outside the US (non-US born) [2]. Identification and treatment of latent tuberculosis infection (LTBI) can prevent the development of TB disease [3]. In Massachusetts, an estimated 190,904 persons (2.9% of residents) live with LTBI, with 84% of infections in non-US born populations [4]. Broad scale-up of LTBI screening and treatment [5] in non-US born individuals is a key strategy in reducing health disparities and achieving TB elimination [6].

The US Preventive Services Task Force recommends screening for LTBI in populations at increased risk as part of routine preventive care [7]. The LTBI care cascade is a challenging multi-step process, from screening through diagnostic testing to treatment. The proportion of US patients who complete LTBI treatment has been observed to be as low as 10% [8], with drop-off at each step along the cascade. Providing patient-centered LTBI care in federally qualified health centers (FQHCs) to non-US born individuals may prevent drop-off and improve treatment completion. Care for LTBI should include standardized screening approaches, testing with an interferon-gamma release assay (IGRA), and treatment with the shortest possible regimen. Establishing an efficient LTBI care cascade at the patient’s medical home, coupled with enhanced epidemiologic surveillance and programmatic public health support could advance TB elimination in the US [9].

Lynn, Massachusetts is a mid-size city north of Boston; 36.7% of 101,253 residents are non-US born [10]. Most of the city falls into the highest quartile of the 2018 Social Vulnerability Index (SVI) across its four component themes [11]. Lynn Community Health Center (CHC) cares for over 43,000 patients: 95% live below 200% of the federal poverty limit, 82% identify as racial and/or ethnic minority, 53% are best served in a language other than English, and 17% are uninsured [12]. During 2012–2016, TB disease incidence among Lynn residents was 10.2/100,000, 340% higher than the statewide incidence.

The Massachusetts Department of Public Health (MDPH) partnered with Lynn CHC to scale up targeted LTBI testing and treatment for their non-US born patients within the CHC, rather than referring these patients to a TB specialty clinic for care. The three-year *TB Elimination through Screening and Treatment* (TBEST) demonstration project sought to reach patients who were not screened through routine public health activities such as refugee medical screening or TB contact investigation.

## Methods

**Fig. 1:**
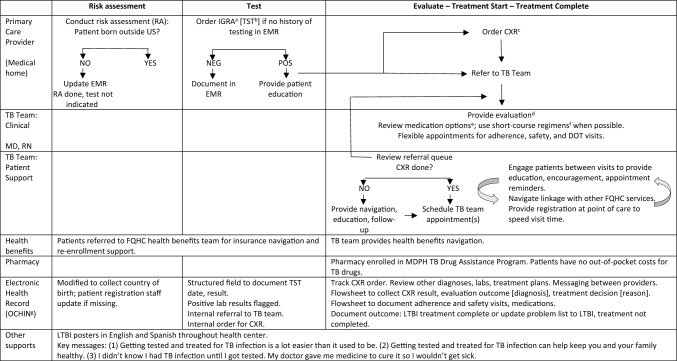
Lynn Community Health Center LTBI care cascade workflow. ^a^QuantiFERON Gold in-tube (QFT-GIT). ^b^Tuberculin skin test. ^c^Chest X-ray; radiology on-site, no appointment required. ^d^See www.cdc.gov/tb/topic/testing/diagnosingltbi.htm for evaluation scope. ^e^See https://www.cdc.gov/mmwr/volumes/69/rr/rr6901a1.htm?s_cid=rr6901a1_w. ^f^Four months self-administered daily rifampin (4R) or isoniazid and rifapentine once weekly by directly observed therapy for 12 weeks (3HP).  ^g^See https://ochin.org

TBEST was implemented in October 2016. The Lynn CHC TB team was composed of two physicians, one nurse, and two community health workers (CHWs). A patient navigator (PN) was added in year two. Project funds supported clinician administrative time and non-clinical staff. Primary care providers (PCPs) and staff across Lynn CHC were oriented to LTBI and TBEST. Patient registration was modified to assess the patient’s country of birth. Health center PCPs were encouraged to screen non-US born patients for TB during routine clinical encounters. All subsequent encounters in the LTBI care cascade also took place at Lynn CHC, supported by the TB team (Fig. 1). CHWs provided LTBI education and addressed barriers to retention in care. PNs assisted with arranging appointments and ensuring access to health benefits.

The MDPH Institutional Review Board determined this project to be non-research.

### Data Collection

The Lynn CHC electronic health record (EHR) was examined for data elements essential to the LTBI care cascade based on a prioritized data dictionary. An EHR flowsheet added by Lynn CHC facilitated the collection of data elements utilized for the public health assessment of the LTBI care cascade. Minor updates were made to the data dictionary and EHR as needs were identified over the course of the project.

### Data Analysis

Data were extracted from the EHR as a comma-separated values (CSV) file, readable by Microsoft Excel; the file was transmitted securely to MDPH bi-weekly. EHR data were processed using SAS v9.4. Surveillance information was uploaded to MAVEN, the MDPH infectious disease surveillance and case management system. Descriptive statistics were compiled, and statistically significant differences were assessed based on comparison to a referent for age group, gender, world region, ethnicity, and race. Lynn CHC project staff reviewed records of those who exited the care cascade at each step and assigned each a reason code for use in analysis of barriers.

LTBI care cascade reports were produced monthly. Quarterly, MDPH and Lynn CHC met with TBEST external evaluators to review performance measures and reflect on successful practices and challenges.

## Results

From October 1, 2016 to September 30, 2019, Lynn CHC PCPs tested 10,319 non-US born patients for TB infection, a 1.9-fold increase over an estimated baseline of 1200 tests/year. To allow a minimum 12-month follow-up, the study cohort consisted of patients with a TB infection test between October 1, 2016 and March 31, 2019 (n = 8827). Most patients (8010, 90.7%) were tested with an IGRA; the remainder (817, 9.3%) underwent tuberculin skin testing (TST). There were 131 countries of birth represented in the cohort, with the largest numbers from Dominican Republic (2163, 24.5%) and Guatemala (1836, 20.8%). Overall, 1368 (15.5%) patients had a positive test for TB infection, with statistically significant differences in rates by age and world region consistent with known TB epidemiology (Table 1).

**Table 1:** Latent TB infection care cascade

Characteristic	Tested: IGRA^a^ or TST^b^	Positive IGRA or TST: n (% positive)	Evaluated: n (% of positive IGRA or TST)	Treatment recommended: n (% of positive IGRA or TST)	Treatment start: n (% of positive IGRA or TST)	Treatment complete: n (% of positive IGRA or TST)
Total	8827	1368^c^	1036 (75.7)	943 (68.9)	835^d^ (61.0)	645^e^ (47.1)
Age (years)						
0–5	265	7 (2.6)^*^	5 (71.4)	5 (71.4)	5 (71.4)	3 (42.9)
6–18	896	48 (5.4)^*^	37 (77.1)	35 (71.1)	35 (71.1)	28 (58.3)
19–25	1246	92 (7.4)^*^	66 (71.7)	62 (67.4)	53 (57.6)	39 (42.4)
26–44^f^	3695	558 (15.1)	420 (75.3)	392 (70.3)	332 (59.5)	253 (54.1)
45–65	2087	468 (22.4)^*^	351 (75.0)	308 (65.8)	288 (61.5)^*^	227 (48.5)
66+	638	195 (30.6)	157 (80.5)	141 (72.3)	122 (62.6)	95 (48.7)
Gender						
Female	5550	813 (14.6)^*^	612 (75.3)	554 (68.1)	477 (58.7)^*^	357 (43.9)
Male^f^	3277	555 (16.9)	424 (76.4)	389 (70.1)	358 (64.5)	288 (51.2)
WHO region						
Africa	900	257 (28.6)^*^	185 (72.0)	172 (66.9)	146 (56.8)	121 (47.1)^*^
Americas	5382	764 (14.2)^*^	616 (80.6)	572 (74.9)	513 (67.1)	391 (51.2)
Eastern Mediterranean	358	74 (20.7)^*^	55 (74.3)	42 (56.8)	38 (51.4)	27 (36.5)
Europe^f^	194	19 (9.8)	13 (68.4)	11 (57.9)	9 (47.4)	5 (26.3)
South East Asia	240	40 (16.7)^*^	33 (82.5)	27 (81.8)	24 (88.9)	18 (75.0)
Western Pacific	518	107 (20.7)^*^	82 (76.6)	72 (67.3)	64 (59.8)	51 (47.7)
Unknown	1235	107 (9.7)	52 (48.6)	47 (43.9)	41 (38.3)	32 (29.9)
Hispanic ethnicity						
Yes	5315	633 (11.9)^*^	508 (80.3)^*^	471 (74.4)	419 (66.2)	324 (51.2)
No^f^	3329	707 (21.2)	510 (72.1)	455 (64.4)	402 (56.9)	311 (44.0)
Unknown	183	28 (15.3)	18 (64.3)	17 (60.7)	14 (50.0)	10 (35.7)
Race (self-reported)						
American Indian/Alaskan Native	189	18 (9.5)	13 (72.2)	11 (61.1)	11 (61.1)	8 (44.4)
Asian	856	162 (18.9)^*^	122 (75.3)	106 (65.4)	95 (58.6)	73 (45.1)
Black African American	2531	627 (24.8)^*^	465 (74.2)	431 (68.7)	380 (60.1)	303 (48.3)
Multi-race	57	11 (19.3)^*^	8 (72.7)	8 (72.7)	7 (87.5)	4 (57.1)
Native Hawaiian/Pacific Islander	142	18 (12.7)	15 (83.3)	14 (77.8)	14 (77.8)	10 (55.6)
White^f^	3285	346 (10.5)	274 (79.2)	245 (70.8)	215 (62.1)	167 (48.3)
Not disclosed/unknown	1767	186 (10.5)	139 (74.7)	128 (68.8)	113 (60.8)	80 (43.0)

^a^Interferon gamma release assay (n = 8010)

^b^Tuberculin skin test (n = 817)

^c^Positive test results by test type: IGRA n = 1312 (16.4%); TST n = 56 (6.9%)

^d^Treatment starts by regimen: four months of rifampin (4R) n = 516 (61.8%); three months of isoniazid and rifapentine weekly (3HP) n = 148 (17.7%); nine months of isoniazid (9H) n = 146 (17.5%); unknown/not documented n = 25 (3.0%)

^e^Treatment completions by regimen: 4R n = 397 (61.6%); 3HP n = 127 (19.7%); 9H n = 107 (16.6%); unknown/not documented n = 14 (2.2%)

^f^Reference group

*P<0.05

Of the 1368 patients with TB infection, 1036 (75.7%) underwent a TB clinical evaluation that included medical examination and chest imaging. Of those, three individuals were diagnosed with TB disease and treated with first-line TB therapy. LTBI treatment was recommended for 943 (68.9%), initiated by 835 (61.0%), and completed by 645 (47.1%). Among patients who completed a full course of therapy, 397 (61.6%) completed four months of rifampin, 127 (19.7%) completed three months isoniazid and rifapentine weekly, and 107 (16.6%) completed nine months of isoniazid. Fourteen patients completed treatment but did not have a regimen documented.

The greatest drop-offs in the care cascade occurred between testing for TB infection and clinical evaluation after a positive test (24.3%) and between the recommendation for LTBI treatment and completion of a treatment course (22.8%). Among the 332 patients not completing clinical evaluation, 51.2% had at least one phone contact with the TB team and 28.9% did not have a referral to the TB team. The most common reasons for treatment non-completion were loss to follow-up (51.1%), medication side effects (29.5%), or patient choice (12.6%) (Table 2). There were few significant differences in treatment recommendation, initiation, or completion among demographic groups once they were evaluated by the TB team (Table 1).

**Table 2:** Reasons for drop-off on the LTBI care cascade and barriers to engagement

Care cascade step	Drop-off reasons		Barriers to engagement	
			System-level	Person-level
Clinical evaluation to establish LTBI diagnosis following positive test for TB infection	332/1368 (24%) patients did not complete medical evaluation after a positive test for TB infection		• Provider messaging about positive test unclear • Centralized referral process • Messaging after “negative” or “normal” chest X-ray • EHR workflow not clear; missed referrals	• Patient perception/lack of clarity regarding diagnosis of TB infection • “Negative” chest X-ray interpreted as no need for further TB management • Not sick • Concerns about radiology • TB stigma
	Contacted at least once by the TB team/Did not keep evaluation appointment	170 (51%)		
	No referral from primary care to the TB team	96 (29%)		
	Patient declined evaluation	14 (4%)		
	Other: Evaluated elsewhere, moved	10 (3%)		
	Unknown reason	42 (13%)		
Treatment recommendation following establishment of LTBI diagnosis	93/1036 (9%) patients completed evaluation, but LTBI treatment was not recommended		• Some degree of over-testing when primary care providers encouraged to test non-US born patients	
	Prior LTBI treatment	55 (59%)		
	TB infection ruled out	5 (5%)		
	TB disease diagnosed	3 (3%)		
	Other/Unknown reason	30 (32%)		
Initiation of LTBI treatment following treatment recommendation	108/943 (11%) patients had LTBI treatment recommended, but did not start treatment		• Screening during pregnancy associated with delay in treatment; will need to re-engage patients post-partum	• Concerns about medications • Uncertainty about TB infection and need for treatment
	Treatment deferred by provider	51 (47%)		
	Patient declined treatment	26 (24%)		
	Other: moved, died	4 (4%)		
	Unknown reason	27 (25%)		
Completion of LTBI treatment course	190/835 (23%) patients initiated, but did not complete, LTBI treatment		• Monthly visits [weekly for observed treatment] • Time required for registration, check-in process each visit • Insurance continuity challenges	• Community beliefs and messaging incongruent with LTBI treatment recommendations • Competing demands • Medication side effects
	Lost to follow-up or stopped on own	121 (64%)		
	Side effects or provider recommendation	61 (32%)		
	Other: moved, died, insurance issue	8 (4%)		

## Discussion

TBEST demonstrates that patient-centered TB services embedded in the FQHC medical home, led by a clinical team and supported by CHWs, can extend targeted testing and treatment to more persons at risk for LTBI. TBEST champions were developed among existing staff at the facility. Low-cost EHR modifications, such as the addition of a flowsheet to collect LTBI data, were implemented by staff and designed to fit within scope of practice. Linkage to MDPH resources, including training, clinical consultation, and TB care cascade data collection, analysis, and reporting further promoted quality improvement and sustainability beyond the demonstration project period.

Patient retention in the LTBI cascade of care is challenging. Systems barriers such as complex navigation can be associated with referral from the site of a positive screening test to a centralized TB clinic for diagnostic evaluation and treatment. In our project, although diagnostic evaluation and treatment were available on-site, drop-off was observed along the care cascade. TBEST strategies included active outreach to patients with positive screening tests to introduce the TB team and assist with navigation. LTBI messaging in primary care was streamlined, and multilingual posters encouraged testing and treatment. For continuing patients, the TB team offered flexible visits and evening hours, waived central registration requirements, and provided LTBI medications with no out-of-pocket costs while offering longitudinal education and interpersonal support to build patient trust. Further study and strategies to improve engagement after a positive screening test are warranted.

This project was designed as pilot intervention to address known, but not previously documented challenges with engagement and retention of a high-risk population in the LTBI care cascade. As a result, the analysis of the care cascade at Lynn CHC was limited by a lack of pre-intervention baseline data, which precluded the establishment of a clear historical control for comparison. Additionally, due to reliance on EHR to track project data and the complexity of extracting the data, it was only possible to track individual patients through the LTBI care cascade using EHR indicators like dates of diagnosis and treatment. Chart reviews of narrative notes regarding patient engagement would provide additional insight into regimen changes and specific adverse events.

The TBEST effort embedded TB care within a FQHC serving a diverse community with a high SVI and a high proportion non-US born individuals—those patients who may be least likely to access referred LTBI care. This represents a refocusing of care delivery, bringing high-quality services closest to the patients with the greatest need and supporting both providers and patients to navigate the LTBI care cascade. Expansion of TB services in FQHCs and other medical homes will require staffing investment and tailoring of the EHR. These efforts, however, offer the potential for higher patient engagement and retention in LTBI care and the prevention of TB disease in high-risk communities, moving the US closer towards TB elimination.

## New Contribution to the Literature

This study of TBEST describes the dynamics of retention in care across the LTBI care cascade for a large and diverse non-US born FQHC patient population at risk for LTBI and not otherwise screened through routine public health activities. We present a summary of the clinical and public health supports that were implemented to establish patient-centered LTBI care at a FQHC. Additionally, we evaluate reasons for drop-off along the cascade. The TBEST project represents a LTBI care quality improvement at the level of the CHC. This demonstration project suggests that decreasing barriers to care for non-US born patients at risk for LTBI is feasible and could be scaled to similar care settings elsewhere.
